# Tfh Cells in Health and Immunity: Potential Targets for Systems Biology Approaches to Vaccination

**DOI:** 10.3390/ijms21228524

**Published:** 2020-11-12

**Authors:** Hannah Law, Vanessa Venturi, Anthony Kelleher, C. Mee Ling Munier

**Affiliations:** 1 The Kirby Institute, UNSW Sydney, Sydney 2052, Australia; vventuri@kirby.unsw.edu.au (V.V.); akelleher@kirby.unsw.edu.au (A.K.); 2 St Vincent’s Clinical School, UNSW Sydney, Sydney 2052, Australia

**Keywords:** T follicular helper cells (Tfh), germinal centre (GC), lymph node (LN), T cell receptor (TCR), systems biology, scRNA-seq

## Abstract

T follicular helper (Tfh) cells are a specialised subset of CD4+ T cells that play a significant role in the adaptive immune response, providing critical help to B cells within the germinal centres (GC) of secondary lymphoid organs. The B cell receptors of GC B cells undergo multiple rounds of somatic hypermutation and affinity maturation within the GC response, a process dependent on cognate interactions with Tfh cells. B cells that receive sufficient help from Tfh cells form antibody-producing long-lived plasma and memory B cells that provide the basis of decades of effective and efficient protection and are considered the gold standard in correlates of protection post-vaccination. However, the T cell response to vaccination has been understudied, and over the last 10 years, exponential improvements in the technological underpinnings of sampling techniques, experimental and analytical tools have allowed multidisciplinary characterisation of the role of T cells and the immune system as a whole. Of particular interest to the field of vaccinology are GCs and Tfh cells, representing a unique target for improving immunisation strategies. Here, we discuss recent insights into the unique journey of Tfh cells from thymus to lymph node during differentiation and their role in the production of high-quality antibody responses as well as their journey back to the periphery as a population of memory cells. Further, we explore their function in health and disease and the power of next-generation sequencing techniques to uncover their potential as modulators of vaccine-induced immunity.

## 1. Introduction

The adaptive immune response relies on the orchestration of a complex network of interactions between antigen, numerous cell types and the signalling and effector molecules they produce. The ability to generate immunological memory is dependent on T cells, and the diversity of the T cell receptor (TCR) repertoire plays a significant role in maintaining the delicate balance between the ability to recognise antigen with extraordinary specificity and the ability to continue detecting an immense range of potential pathogens. Ideally, T cell responses would always establish sterilising immunity, but realistically, the obstacles of antigenic variability, pathogen evasion strategies and T cell exhaustion mean total elimination of the pathogen may not be possible.

Vaccination is undoubtedly one of the most successful interventions for reducing the burden of infection-related disease on communities, and it is responsible for significant decreases in disease prevalence, morbidity, and mortality globally [1]. Establishing immune memory to prevent the spread of pathogens throughout communities is the ultimate goal of vaccination. This is achieved during the germinal centre (GC) reaction, where the primary function is to establish and maintain memory cell populations that can mediate rapid recall responses and prolonged production of antibodies [2]. Of particular interest in the GC reaction are the interactions between Tfh cells and GC B cells and how the relationship between these cell populations could be leveraged to improve immune responses to vaccination. 

The study of the differentiation and functioning of Tfh cells has demonstrated that dysregulation in these processes can drive inadequate immune responses and can be linked to autoimmunity. It is therefore crucial to understand the development and complex functioning of Tfh cells to potentially prevent or counteract their dysregulation and for generation of rational vaccine design. In this review, we discuss the differentiation of naïve T cells into fully mature Tfh cells, the complex interactions between Tfh and B cells in the GC and the role of circulating Tfh (c-Tfh) cells in the periphery during health and disease. In addition, we discuss the contribution of next-generation sequencing techniques to our understanding of other immune cell biology and the potential future applications of these technologies in uncovering insights into Tfh cell responses to infection and vaccination.

## 2. Role of the T Cell Receptor

The ability to maintain immune homeostasis, control continuous, multiple insults to the immune system and generate immunological memory is dependent on T cells. Defence against numerous pathogens, allergens and tumours can be attributed to the generation of a highly diverse repertoire of TCRs during cell development in the thymus [3,4]. Each T cell possesses a TCR that enables response to an antigen with extraordinary specificity whilst retaining enough heterogeneity as a population to support recognition of the immense diversity of antigens. The TCR expressed on a cell surface also plays a role in self-tolerance, cell lineage and cell fate decisions. Each TCR has a unique antigen-binding site formed by three complementarity-determining region (CDR) loops, which engage with the peptide–major histocompatibility complex (pMHC) molecule and determine the antigen(s) bound to the surface of antigen-presenting cells (APCs) that the T cell will respond to [5,6]. In addition to the combinatorial diversity achieved through pairing of different variable (*V*), diversity (*D*) and joining (*J*) genes, the enormous diversity of the TCR repertoire is further enhanced by the variation in the amino acid sequence at the primary antigen-binding site, termed the CDR3 [7,8,9]. Although only a small set of genes encode for the TCR, recombination of non-contiguous sequences and deletion and insertion of nucleotides increase the number of potential TCRs to between 10^15^ and 10^20^ clonotypes [10,11]. However, the realistic possible diversity of the repertoire is limited by the ~10^12^ T cells within the human body [12]. One study utilised high-throughput sequencing to determine that the thymic repertoire of αβ T cells ranged from 40 to 70 × 10^6^ and 60 to 100 × 10^6^ unique β and α sequences, respectively [13]. Therefore, the ability of one TCR to recognise and effectively respond to more than one antigen, a concept termed cross-reactivity, is essential to our ability to mount immune responses against the large potential diversity of pathogens [14]. Characteristics of the repertoire, including TCR affinity, TCR avidity, clonality and breadth, all play substantial roles in our ability to identify and respond to the immense diversity of often highly variable antigens (reviewed in [4]). Therefore, although it is evident that specificity and diversity can be achieved, the balance between TCR frequency, cross-reactivity and diversity is essential to optimum immune surveillance.

## 3. Lymphocyte Development in the Thymus

**Figure 1:**
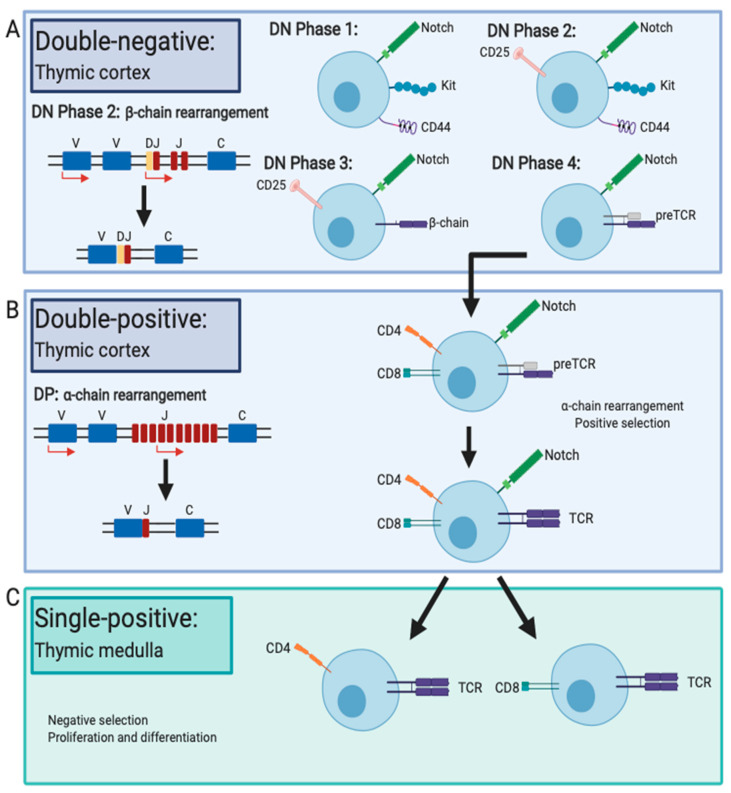
T cell differentiation in the thymus. (**A**) Immature thymocytes in the thymic cortex upregulate Notch, Kit and CD44 to begin differentiation in double-negative (DN) phase 1. As CD25 expression is upregulated, thymocytes progress into DN phase 2, where T cell receptor (TCR) β-chain rearrangement begins. Rearrangement of the β-chain locus of the TCR, from the Diversity β (*D*β) to the Joining β (*J*β) region, begins, and as expression of CD44 and Kit are reduced, *V*β to *D*β*J*β rearrangements occur and cells progress into the DN3 phase. Only cells that successfully produce a reproductive β-chain survive and progress into DN phase 4, where the β-chain is paired with a surrogate pre-α chain to form the pre-TCR. (**B**) During the DN4 phase, expression of CD4 and CD8 are triggered, progressing cells into the double-positive (DP) phase of differentiation. It is here that α-chain rearrangement begins, and recombination of *V*α to *J*α gene segments continues until cell death or positive selection occurs. During the DP phase, cells produce a functional TCR and undergo positive selection so that ultimately only functionally competent and self-tolerant cells remain. (**C**) Thymocytes then migrate into the thymic medulla, where downregulation of Notch and either CD4 or CD8 occurs. This is the final phase of differentiation, termed the single-positive phase, where naïve T cells expressing either CD4 or CD8 and a functional TCR can exit the thymus and traffic to the periphery (created with BioRender.com).

The stages of differentiation and functional maturation of thymocytes are marked by rearrangement of the TCR genes, expression of the TCR and expression of cell surface proteins. Interaction with the thymic stroma initiates commitment to and differentiation along the T cell lineage pathway. The initial phase of thymocyte differentiation is termed the double-negative (DN) phase due to the absence of expression of the T cell coreceptors CD4 and CD8 (Figure 1A). Naïve DN1 thymocytes express Notch, Kit and CD44, but as they progress towards mature thymocytes, surface expression of the IL-2Rα chain, CD25, is initiated, at which stage they are called DN2 cells [15]. During the DN2 phase, sustained Notch expression is required for rearrangement of the β-chain locus of the TCR, from the Diversity β (*D*β) to the Joining β (*J*β) region, to begin [16]. As expression of CD44 and Kit are reduced, *V*β to *D*β*J*β rearrangements occur, and cells progress into the DN3 phase [17]. Productive expression of a rearranged β chain paired with a surrogate pre-T cell receptor α chain to form the pre-T cell receptor (pre-TCR) is essential to progression through the DN3 phase [18,19,20]. If cells fail to produce successful rearrangements of the β locus, they do not progress and eventually undergo apoptosis. DN3 thymocytes then lose CD25 expression and progress to the final DN phase, DN4, where the pre-TCR induces ligand-independent dimerisation, causing the cell to begin to proliferate and express both CD4 and CD8 on their cell surface as cells enter the double-positive phase of differentiation (Figure 1B) [17].

Primary and secondary recombination of *V*α to *J*α gene segments occurs during this double-positive phase, estimated to take an average of five rounds of recombination per allele until positive selection or cell death occurs [21,22,23]. During this double-positive phase, thymocytes undergo positive selection, where ~98% of thymocytes are eliminated [24,25]. Negative selection in the thymic medulla ensures that T cells with high self-pMHC reactivity undergo apoptosis, eliminating risk of autoimmunity [26,27]. Paradoxically, weak self-pMHC recognition is a requirement for development of self-tolerance in the thymic cortex and ultimately T cell survival [28]. T cells with low self-pMHC recognition are rescued, and T cells with no affinity of self-pMHC undergo apoptosis, a process termed positive selection [29]. The remaining ~2% of thymocytes selected as both functionally competent and self-tolerant progress to the final stages of differentiation, downregulating either CD4 or CD8 and becoming single-positive T cells that exit the thymus as naïve T cells (Figure 1C). Naïve T cells migrate to the periphery and subsequently traffic to lymphoid tissues throughout the body.

## 4. T Cell Entry into the Lymph Nodes

Lymphocyte trafficking and entry into the lymph nodes (LN) is made possible by an extensive network of lymphatic capillaries and vessels that infiltrate tissues within the body [30]. Lymphocytes contained in the lymph drain from interstitial spaces into the afferent lymphatic vessels (LVs), where their migration into the draining LN is mediated by interactions of C-C motif chemokine ligand 21 (CCL21) expression on vessels and C-C chemokine receptor type 7 (CCR7) expressed on T cells [31,32]. Lymphatic endothelial cells that line the LVs secrete sphingosine 1-phosphate (S1P), which contributes to T cell LN migration via S1P receptor 1 binding [33,34,35]. In contrast, lymphocytes in the blood drain into LNs via high endothelial venules (HEVs), a process initiated by CD62L (L-selectin), which facilitates rolling and tethering of lymphocytes to the walls of venules [36]. Further, migration of naïve T cells through HEVs is mediated by a complex adhesion cascade facilitated by interaction between CCL21 and CCR7 [37]. Once inside the LN, the fibroblastic reticular cell (FRC) network creates a scaffold-like architecture and a CCL21 gradient that influences dendritic cells (DCs) and T cells intranodal positioning and migration through the LN [38,39].

## 5. Tfh Cell Migration through the Lymph Nodes

**Figure 2:**
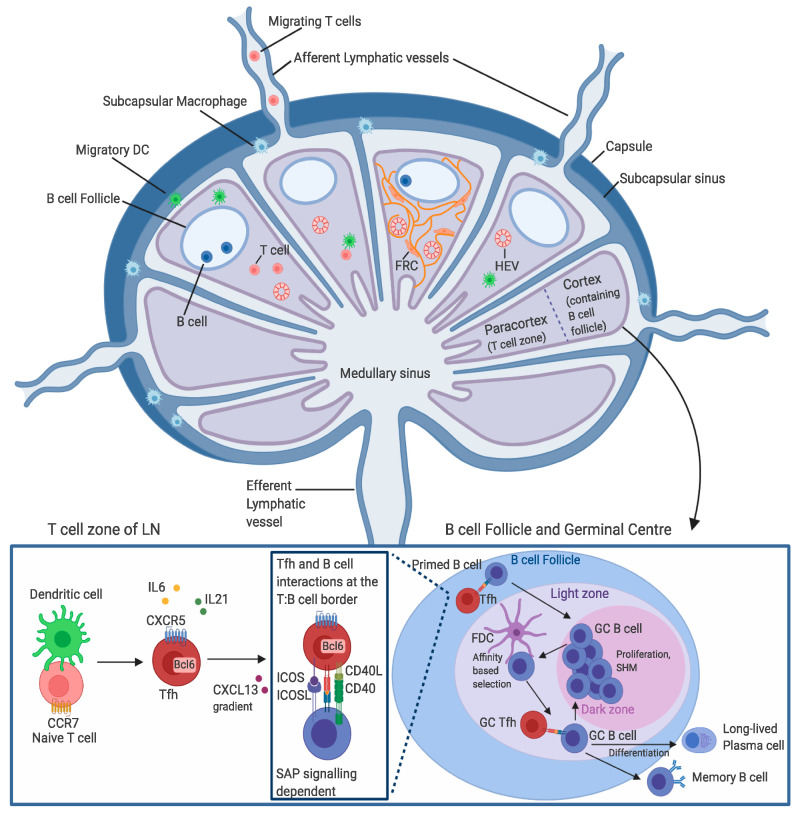
Tfh circulation in the lymph node (LN). Naïve T cells enter the LN either through afferent lymphatic vessels, or from the blood via the high endothelial venules (HEV). A complex adhesion cascade driven by interactions of CCR7 and CCL21 facilitate naïve T cell migration through the medulla and to the paracortex (T cell zone). Here, dendritic cells (DCs) prime naïve T cells, resulting in CCR7 downregulation and CXCR5 upregulation. CXCR5 expression promotes Tfh cell differentiation and migration to the border of the B cell follicle via a CXCL13 gradient. It is here, at the T–B cell border, that cognate interactions involving critical signalling through ICOS/ICOSL, CD40L/CD40, TCR/MHC-II and SAP/SLAM support further Tfh cell polarisation and differentiation, B cell help and germinal centre (GC) formation. The GC reaction begins with Tfh and B cell interactions, where B cells will either differentiate into short-lived antigen-secreting cells or enter the GC dark zone (DZ) to undergo multiple rounds of somatic hypermutation (SHM), selection and proliferation. B cells then exit the DZ and migrate into the light zone (LZ) of the GC, where they compete for antigen presented by follicular dendritic cells (FDC). Tfh cells then selectively provide help to B cells possessing high-affinity B cell receptors (BCRs). After these Tfh–B cell interactions, B cells either differentiate into memory B cells, differentiate into long-lived plasma cells or re-enter the DZ for further rounds of SHM and selection (created with BioRender.com).

The structural components and major cell migration pathways are schematically summarised in Figure 2. Lymph and its cellular components traffic through the subcapsular sinus and medullary sinuses of lymph nodes. Here, T cells survey subcapsular macrophages and dendritic cells in the T cell zone for cognate antigen (Figure 2), which, if found, will promote entry into the LN parenchyma [40]. Solubility and size of an antigen can impact the stimulation process as different size/forms of antigen, such as soluble or particulate, are directed to different microanatomical sites within the LN (reviewed in [41]). For example, soluble antigens are taken up and sequestered by lymphatic endothelial cells. Antigens of low molecular weight are channelled via the conduit system surrounded by FRCs, ultimately delivering antigens to follicular DCs in the B cell follicle, allowing cognate B cell sampling of antigen [42,43]. Immune complexes and particles traverse the subcapsular sinus via help from subcapsular macrophages, where they then interact with FDCs and B cells [40,44]. If a cognate antigen is not identified via TCR–pMHC-II interactions, T cells will transmigrate to the medullary sinuses and exit the LN via the efferent LVs [45]. The innermost section of the LN, termed the medulla, is the site where the positioning of DCs determines interaction with antigen and subsequent contact with T cells [46]. Once T cells have encountered antigen, migration towards the paracortex or the T cell zone begins, and CCR7 expression is downregulated, accompanied by C-X-C chemokine receptor 5 (CXCR5) upregulation (Figure 2) [47]. This promotes homing to the B cell follicle of the LN cortex, formation of the GC and differentiation of Tfh and GC B cells. Following stimulation, memory cells formed during the GC reaction home to particular sites within the lymph node, where they await reactivation. One such population is memory Tfh cells, which preferentially migrate to the T–B cell border in the LN cortex via upregulation of CXCR5 [48]. It is here that, upon reactivation, these long-lived memory Tfh cells provide help to B cells, and a subset further differentiate into effector Tfh cells by Bcl6 upregulation [48]. Importantly, the lymphatic flow of the subcapsular sinus plays an essential role in secondary immune responses as an efficient transport mechanism for follicular memory T cells to migrate out of the GC and survey APCs, enabling proliferation and necessary rapid responses to antigen re-encounter [49].

## 6. T Cell Egress from the Lymph Node

Similar to the role of S1P in the recruitment and trafficking of cells into the LN, a S1P gradient created by low concentrations in the LN and high concentrations in the lymph causes the transmigration of T cells into the lymphatic sinuses and exit via the efferent LVs [50]. The major homeostatic cellular component of efferent lymph is CD4+ T cells; however, antigen presence and stimulation have been shown to influence efferent lymph composition. Following antigen stimulation, the LN will undergo three transitory states until returning to homeostasis. Initially, lymphocyte output is decreased as the LN enters quiescence. This is followed by an intermediary increase in lymphocyte levels to above resting levels in the lymph and finally an ultimate reduction in lymphocyte output to homeostatic levels [51].

## 7. Stages of Tfh Cell Differentiation

Tfh cell differentiation is a multistage and complex process involving many signalling pathways [52]. Unlike the differentiation pathways of other specialised CD4+ T cells, Tfh cell differentiation accommodates significant heterogeneity within the population [53]. Initially, naïve CD4+ T cells are primed by DCs in the T cell area of the LN, resulting in downregulation of CCR7 and increased expression of CXCR5, which is essential for Tfh cell homing to the B cell follicle (Table 1) [54,55,56]. It has been shown that during this early phase of Tfh differentiation, interleukin (IL)-6-mediated induction of the transcription factors signal transducer and activator of transcription (STAT) 1 and STAT3 is required for Bcl6 induction and downregulation of the IL-2Rα, respectively, limiting Th1 differentiation and promoting Tfh differentiation [57,58,59]. IL-21-dependent expression of inducible costimulator ligand (ICOSL) by B cells and its essential interactions with ICOS are well established as regulators of humoral immunity [60]; however, IL-21 can also regulate Tfh differentiation through the transcription factor c-Maf [61]. Regulation of the expression of cytokines, including IL-21 and IL4, that promote B cell and Tfh cell differentiation and proliferation is the major role of c-Maf expressed by Tfh cells [62,63]. Interestingly, dendritic cell production of IL-12 induces sustained expression of ICOS and has been demonstrated to be essential for early expression of, and closely correlated to, levels of CXCR5 expression [64,65,66]. Upregulation of CXCR5 is accompanied by downregulation of CCR7 and the transcription factor Blimp-1, mediated by upregulation of KLF2 [67,68]. Also critical to cell fate and function is higher TCR affinity, resulting in longer TCR–MHC-II dwell time [69,70,71]. Immature Tfh cells subsequently follow a gradient of CXCL13, the chemokine ligand for CXCR5, to migrate towards the T–B cell border [72,73]. This is where they interact with B cells and commit to the Tfh lineage.

The later phases of Tfh differentiation highlight the symbiotic relationship between Tfh cells and B cells, which are essential to the survival of maturing Tfh cells, providing additional signals aiding in the maintenance of Bcl6 expression and their development into functional B cell helpers. The T–B cell border within the LN is an important site where signalling between signalling lymphocytic activation molecule (SLAM) family receptors expressed on T and B cells occurs [74]. Interactions between SLAM expressed on B cells and SLAM-associated protein (SAP) expressed on T cells are necessary for forming stable T–B cell conjugates [75]. Signalling interactions between CD40L expressed by Tfh cells and CD40 expressed on B cells are essential to the formation and maintenance of GCs as well as promoting class switching of antibodies, inhibiting plasma cell differentiation and providing crucial GC B cell survival signals [76,77,78,79]. Therefore, both GC formation and T cell-dependent antibody responses are contingent on these interactions [74,75]. Furthermore, studies have demonstrated that for persistent T cell migration to the T–B cell border, normal Tfh cell development and optimal germinal centre responses as well as engagement of ICOS via ICOSL on follicular bystander B cells is essential [80]. Blockade of ICOSL function ultimately results in Tfh cell and GC formation inhibition [64,81]. This is further supported by findings that signalling via ICOS promotes the expansion of Tfh cell populations and overexpression results in spontaneous Tfh cell and GC development [82,83]. Deenick and colleagues (2010) suggested that Tfh cell development is more heavily influenced by the role of ICOS/ICOSL interactions in the positioning of B cells to provide plentiful sources of antigen rather than co-stimulation [84]. Therefore, ICOS/ICOSL interactions play an important role in T and B cell positioning within the follicle as well as co-stimulation.

Full differentiation of Tfh cells occurs within the GC, the site where their primary role in the development of an immune response is performed [52,85]. GC Tfh cells are characterised by their high expression of CXCR5 and low CCR7, high ICOS, high PD-1 and high IL-21 expression [86]. In addition, SAP is highly expressed on GC Tfh, where it is essential in sustaining long-lasting T–B cell adhesion [87,88]. SAP plays a critical role in preventing robust inhibitory signalling through SLAMF6 by outcompeting Src homology 2 domain-containing protein tyrosine phosphatase 1 (SHP-1) binding [89]. When SAP is bound to SLAMF6, adhesion and development of Tfh cell and B cell responses are supported [90,91]. The importance of these interactions is supported by the observations of severely reduced humoral immunity in X-linked lymphoproliferative patients caused by mutations in the gene *SH2DLA* that encodes SAP [92,93,94].

During the primary immune response, Tfh cells were found to locate to two anatomically distinct compartments of the LN, the follicle mantle (FM) and the GC, within the cortex [95]. FM Tfh and GC Tfh were found to not only be spatially separated but also represented molecularly distinct subpopulations with little migratory crossover [95]. GC Tfh cells expressed higher levels of genes associated with Tfh cell differentiation and proliferation and B cell class switching [95]. FM Tfh cells expressed high amounts of genes associated with temporospatial guidance, cell adhesion and immune regulation [95]. Interestingly, the GC has been described as an open structure in secondary immune responses [86], where migration of Tfh cells between neighbouring GCs and the FM demonstrated a heterogeneous distribution of these subpopulations and therefore greater diversity of Tfh cell help [95], hypothesised to ultimately improve recall responses. Finally, the migration of Tfh into the subcapsular sinus to survey APCs provides an opportunity for antigen-experienced Tfh to egress from the LN and enter circulation, contributing to the c-Tfh cell population. 

## 8. The GC Response and Tfh Cell Function in the Immune Response

Effective humoral immunity is often mediated by sterilising or broadly neutralising antibodies (bAbs), which are produced by memory B cells during the germinal centre reaction [97,98]. The GC forms when antigen is presented by DCs, promoting differentiation and expansion of Tfh cells. GCs are also the site where activated B cells capture and process antigen for presentation on MHC class II complexes [99]. After Tfh cells recognise cognate peptide, further CD4+ T cell differentiation into Tfh cells and B cell differentiation is re-enforced and promoted [100]. Once these initial T–B cell interactions occur, B cells will either differentiate into short-lived antibody-secreting cells (ASCs), or they will enter the GC reaction and undergo further rounds of selection, differentiation and proliferation [97] (Figure 2). 

The GC comprises two functionally distinct compartments (Figure 2): the light zone (LZ) and the dark zone (DZ). In the DZ, B cells undergo multiple iterations of proliferation and somatic hypermutation to produce a heterogeneous B cell population with diverse B cell receptor (BCR) sequences [101]. B cells then exit the DZ and migrate into the LZ, where they compete for antigen bound to the surface of DCs [102,103]. Here, Tfh cells selectively provide help to B cells with high-affinity BCRs due to their ability to internalise and therefore present more antigen to Tfh cells [104,105,106]. After interacting with Tfh cells in the LZ, B cells have three potential fates: (1) differentiate into memory B cells and exit the GC [107], (2) differentiate into long-lived plasma cells and thus exit the GC [108], or (3) re-enter the DZ for further rounds of somatic hypermutation and selection [109]. Many studies have reported this bidirectional movement of B cells between LZ and DZ within the GC [110,111] and suggest that the strength of the interaction between Tfh cells and B cells directly determines B cell fate [97,112]. Interestingly, one study has reported that the proportion of Tfh cell help provided to GC B cells directly translates to the degree of mutations in the B cell receptor, and thus the number of cell divisions, that a given GC B cell will undergo in a single round of selection [113]. Therefore, the GC reaction, preferential support of high-affinity B cells and subsequent production of diverse B cell repertoires are all dependent on help from Tfh cells, although perhaps not to an equal degree as antigen, ultimately impacting on the quality of the immune response.

## 9. Utilisation of c-Tfh Cells to Study Disease States

Tfh cells are empirically defined by their ability to migrate into the GC of secondary lymphoid tissue and the aid they provide to B cells within these anatomically protected sites during the immune response. This establishes an inherent, yet significant, obstacle to studying their role in host responses to infection, vaccination and autoimmunity. Coupled with confounding ethical concerns with accessing healthy human lymph nodes, many studies have utilised animal models, human tonsillar tissue or a circulating, phenotypically similar counterpart, c-Tfh, to highlight the importance of Tfh cell assistance and functioning in the generation of effective immunity. Several studies have utilised CD4+ CXCR5+ ICOS+ c-Tfh as a surrogate biomarker of humoral immunity under the hypothesis that these cells are memory Tfh that egress from the LN following antigen encounter. This hypothesis has been supported by the detection of tetanus-toxoid-specific c-Tfh cells in peripheral blood even years after receiving booster vaccination [114]. Importantly, more recent studies have established the clonal relationship between GC Tfh and their memory c-Tfh counterparts by TCR repertoire sequencing of matched human blood and tonsillar samples [115,116]. Perhaps the most definitive evidence that GC Tfh exit the LN and enter the periphery to form c-Tfh was provided by an interesting study that demonstrated efferent lymph collected from the thoracic duct in humans was enriched for Tfh cells, and treatment with the S1PR1 modulator, fingolimod (FTY720), prevented LN egress and reduced c-Tfh cell numbers and frequency in peripheral blood [117].

Aberrant Tfh cell development and functioning can drive autoimmunity and have been linked with inadequate immune responses [118,119]. However, due to the difficulty accessing bona fide GC Tfh, the use of c-Tfh as a surrogate biomarker has become standard across studies of autoimmunity, immunodeficiencies, allergy and malignancies. In the case of systemic lupus erythematosus (SLE), increases in the frequency of activated c-Tfh positively correlated with serum autoantibody titres and disease severity [120,121,122,123]. Furthermore, elegant studies performed in mice demonstrated that excessive Tfh cell responses and subsequent GC formation were sufficient to cause lupus-like autoimmunity [124,125]. 

Tfh cell dysregulation leading to impaired B cell responses and ultimately humoral immune responses have also been reported in human immunodeficiency virus/simian immunodeficiency virus (HIV/SIV) [126]. Studies have shown that expanded Tfh cell populations harbour higher levels of HIV or SIV DNA compared to other CD4+ T cell subsets, can effectively support productive HIV infection and form a major viral reservoir [127,128,129]. Additionally, expansion of HIV-specific GC Tfh cells and c-Tfh cells have been observed in chronic infection and are associated with increased IL-21 secretion and polyclonal hypergammaglobulinemia [130]. HIV-specific c-Tfh demonstrated a Th-1-like phenotype and functionality, which had a significant positive association with the size of the translation-competent viral reservoir [131]. Interestingly, Th-1-like c-Tfh have been shown to largely lack the ability to help naïve or memory B cells [132]. Conversely, IL-21-producing c-Tfh were observed to have greater helper capacity to induce B cell maturation and class switching in HIV elite controllers and were associated with antigen-specific B cells in HIV progressors [133]. However, significant impairment of c-Tfh cell functioning in chronic HIV infection leads to reduced B cell responses, a phenomenon mirrored in secondary lymphoid organs [134,135]. 

The presence of Tfh is required for IgE production and development of an allergic response [136,137]. Increased numbers of c-Tfh were observed in atopic dermatitis patients, which correlated with activated memory B cells, IgE production and disease severity [138,139]. In allergic rhinitis and asthma, levels of c-Tfh cells with a Tfh2 phenotype are also elevated, correlating with total IgE levels and therefore contributing to a pro-inflammatory milieu in the latter [140]. Further supporting the role of Tfh in allergy, mice with a mutated IL-6R were unable to expand Tfh cell populations, resulting in significantly reduced IgG1 and IgE responses to house dust mite sensitisation when compared to wild-type mice [141]. Therefore, it is evident that the accumulation of c-Tfh has been linked to increased IgE production and proallergic function. Interestingly, these results demonstrate that Tfh dysregulation can lead to impaired B cell responses and humoral immunity across many pathological modalities. 

## 10. Role of Tfh Cells in Vaccination

Multiple studies have demonstrated the emergence of a transient population of PD-1+ICOS+ c-Tfh cells following vaccination with inactivated influenza vaccine [142,143,144,145]. Inactivated influenza vaccine-induced c-Tfh cells were phenotypically similar to their GC counterparts, expressing various activation markers, including PD-1, CD38, ICOS and Ki67 [143,144,146]. Levels of c-Tfh cells correlated with increased Ab serum titres and presence of ASCs and corresponded to peak GC responses in mice and plasmablast response in humans [142,143,144,145,147]. Subsequent studies also demonstrated a positive correlation between activated c-Tfh and an increased affinity of inactivated influenza vaccine-induced Abs and magnitude of memory B cell responses [145,148]. c-Tfh occurrence correlated with active Tfh cell differentiation in secondary lymphoid organs of mice [147], which was mirrored in a vaccination study with an MF59-adjuvanted inactivated H5N1 vaccine [149] and supported by observations of an association between diminished c-Tfh and reduction in vaccine-specific Ab levels in serum of older people in response to vaccination [150]. Secondary immunisation elicited a predominantly plasmablast response, with the induction of few secondary GC responses in mice [151] and in rhesus macaques, although this is potentially attributable to the short interval between immunisations [152]. Further, rhesus macaques that produced higher levels of neutralising Abs had larger GC responses, and antigen-specific CD4+ T cells isolated from peripheral blood were enriched for Tfh-associated genes, including IL-21, ICOS and CD40L [152]. In a subsequent study, GC B cell and antigen-specific CD4+ T cell numbers in the draining LN increased significantly by day 7 post primary vaccination, and this response was 10-fold larger when immunised subcutaneously compared to intramuscularly [153]. However, there was no significant difference in serum IgG titres between the two immunisation protocols, suggesting the magnitude of response may not be directly proportionate to quality of response.

Two critical studies in Tfh cell immunology were published in 2017 that utilised TCR β chain repertoire sequencing to characterise the c-Tfh response to vaccination and establish the clonal relationship to GC Tfh [116,143]. The initial clonal-activated c-Tfh cell response correlated with plasmablast responses and vaccine-specific serum IgG. This was observed following successive seasonal influenza vaccinations and was clonally related to tonsillar GC Tfh [116,143]. Interestingly, between vaccinations, these c-Tfh clones were found in the “inactivated” c-Tfh repertoire and were seen to expand into activated c-Tfh upon antigen re-encounter [143]. These observations were mirrored in an antigen-specific c-Tfh population [116]. A subsequent study observed a transcriptional and clonal relationship between c-Tfh and lymph node GC Tfh [154]. Interestingly, they demonstrated that the combination adjuvant glucopyranosyl lipid adjuvant-stable emulsion (GLA-SE) promoted the emergence of public TCR β sequences as well as long-lived Ab responses [154]. Further to establishing that GC Tfh and c-Tfh are clonally convergent, it has been shown that although circulating non-Tfh cells share few TCR β clones with GC Tfh, they are clonally distinct from c-Tfh cells [115]. A population of Tfh cells isolated from human thoracic duct lymph were shown to be phenotypically and transcriptionally similar to GC Tfh [117]. Utilisation of next-generation sequencing by these recent studies have allowed critical analysis of c-Tfh cell kinetics in response to vaccination but also validation of the hypothesis that c-Tfh cells represent a circulating memory pool critical in aiding B cells during immune responses. 

## 11. The Future Role of Systems Biology Approaches in Characterising Tfh Cells

Next-generation sequencing technologies have played an increasingly important role in understanding Tfh cell biology in recent years [115,116,117,143,154] by enabling researchers to obtain data sets that are sufficiently representative of immune cell populations. Moreover, the recent progress that has been made using such technologies to characterise other immune cell populations and their responses to infection and vaccination indicate strong potential for gaining similar insights for Tfh cells from future studies. 

High-throughput sequencing technologies allow for the large-scale “bulk” sequencing required to capture data for large and/or highly diverse populations of cells. Investigations of the enormously diverse immune receptor repertoires of T cells and B cells have flourished with this technology, yielding valuable immunological insights. For example, the immune receptor repertoires of cells involved in responses to infection or vaccination to pathogens such as Cytomegalovirus (CMV), Epstein-Barr Virus (EBV), Tuberculosis (TB) and influenza have been well characterised, enabling the identification of immune receptor “signatures” in T cells [155,156,157] and B cells [158]. Furthermore, it has been demonstrated that such features may allow prediction of the antigen specificity of cells using machine learning approaches [155,156,157]. Bulk TCR repertoire sequencing has also revealed broad heterogeneity of CD4+ T cell functionality in response to microbial infection and vaccination [159], and transcriptomics have demonstrated that T cells defined as the same clone by their TCR sequence can represent phenotypically distinct subsets [160]. Another application of TCR deep sequencing is as a predictor of disease outcome or state (reviewed in [161]); for example, TCRβ oligoclonality is linked to clinical relapse in juvenile idiopathic arthritis [162], and EBV-cross-reactive TCR clonotypes are enriched in cerebrospinal fluid from multiple sclerosis patients [163].

Single-cell sequencing technologies have also experienced rapid development and growth in application to immunological studies, with increasing capability to generate multi-omic data by measuring the proteome, genome, transcriptome, methylome and spatial expression of single cells [164]. Single-cell RNA sequencing (scRNA-seq) techniques have, to date, been the most popular and have been deployed to analyse the network of cell types, the cytokines and chemokines they produce, their transcriptomic profiles and their involvement in complex immune responses [165]. Explorations of immune responses to vaccination have provided valuable insights by assessing for proteomic and transcriptomic predictors of improved vaccination response. Notably, researchers have identified certain gene expression profiles, including the B cell growth factor tumour necrosis factor receptor superfamily member 17 (TNFRS17) and eukaryotic translation factor 2 alpha kinase 4, that respectively predicted neutralising antibody titres and antigen-specific CD8 T cell responses to yellow fever vaccination [166]. Similar utilisation of systems biology has been applied to groups of interest, such as older adults, where one study validated the identification of genes, including *CAMK4*, previously associated with influenza vaccine responses [167]. New genes of interest that were not previously associated with responses to influenza vaccination and previously uncharacterised genes were also linked to vaccine-induced immunity [167]. Gene signatures identified as possible regulators of vaccine-induced immunity have been described across successive years and multiple cell populations post influenza vaccination [168,169,170], measles vaccination [171] and meningococcal vaccination [172]. The use of single-cell RNA-seq enabled identification of molecular signatures that underpin relationships between Th1 and Tfh cells and showed each cell population differentiated from a highly proliferative single precursor cell during malaria infection in mice [173]. 

Despite the incredible contribution of next-generation sequencing techniques to the understanding of immune responses to infection and vaccination, the discovery of new relationships between cell types and the identification of correlates of protection, there are a number of challenges associated with these technologies [165]. Thus, ongoing development of innovative experimental technologies is focussed not only on increasing throughput and resolution and decreasing costs but also on improving data quality and reproducibility. Also crucial to addressing these challenges is the development of novel computational methods to process, analyse and interpret experimental data. Computational methods utilising machine learning and artificial intelligence approaches is another rapid-growth research area with enormous implications for improving predictive power and developing integrated models of immune responses to infection. Such studies rely on application of these methods to large-scale data sets and thus greatly benefit from recent growth in the number and size of data repositories that facilitate sharing of data. Collectively, these technological advances, coupled with increasing research resources, are allowing researchers to pursue systems biology approaches that are expected to lead to a better understanding of not only Tfh cells but the complex immune system as a whole and how it may best be manipulated to achieve positive health outcomes. 

## 12. Concluding Remarks

Over the past decade, great efforts to characterise the roles and responsibilities of Tfh cells in immunity and infection have yielded interesting and powerful insights, establishing them as key players in immune responses to vaccination in both secondary lymphoid organs and in the periphery and as potential correlates of protection. Despite these significant advances in understanding of the role of Tfh cells in health and immunity, particularly in the field of vaccinology, there are still areas to be explored. Validation of the clonal relationship present between the two hugely heterogeneous populations of cTfh and secondary lymphoid organ (SLO)-derived Tfh is essential to the utilisation of cTfh as a surrogate biomarker in future studies. Could the adoption of scRNA-seq technologies provide powerful insight into the transcriptional factors and signalling pathways that regulate T cell egress from the LN and contribute to our understanding of the relationship between LN Tfh and their circulating counterparts? Ultimately, validation of a clonal relationship will address significant ethical and practical obstacles in accessing and studying SLO-derived Tfh. Given the substantial heterogeneity within the Tfh population, is it possible to identify subpopulations with greater helper functionality or certain gene expression profiles that could be leveraged to improve immune responses to vaccination? Are the Tfh cell responses studied using vaccination as a model truly indicative of their role during the course of natural infection? Finally, there remains a unique opportunity to harness emerging tools to characterise the Tfh cell response to vaccination on a transcriptomic and proteomic level. The ability to identify gene signatures associated with improved vaccine-induced immunity in Tfh cells could be a very powerful tool in rational vaccine design.

## Figures and Tables

**Table 1:** Key Tfh cell receptors and ligands.

Receptor/Ligand	Corresponding Gene	Reciprocal Receptor/Ligand	Function
CCR7	*CCR7*	CCL21	Promotes naïve T cell homing to the T cell zone (paracortex) of the LN [31,47,54,56].
CXCR5	*CXCR5*	CXCL13	Essential for T and B cell homing to the B cell follicle, within the cortex of the LN [55,72,73].
Bcl6	*BCL6*	-	Master transcription factor for Tfh cell lineage. Limits Th1 differentiation through Blimp-1 pathway and supports Tfh cell differentiation [96].
IL-21	*IL21*	IL-21R	Regulates ICOSL expression on B cells essential for Tfh and B cell interactions through ICOS/ICOSL signalling [60]. Regulates Tfh differentiation through c-Maf promotion of IL-21 production [61].
ICOS	*ICOS*	ICOSL	Interacts with ICOSL expressed on B cells, influencing Tfh and B cell positioning within the LN [84]. Necessary for persistent T cell migration to the T–B cell border of the B cell follicle and optimal germinal centre responses [80].
TCR	*-*	MCH-II	T cell receptor that binds with peptide–MHC-II complexes. Higher TCR affinity results in longer TCR–MHC-II interactions, ultimately influencing cell fate decisions [69,70,71].
SAP	*SH2D1A*	SLAMF6 and SLAMF5	Necessary for forming stable T–B cell conjugates via SLAMF6 binding within the GC [75]. Supports Tfh cell adhesion and development and B cell responses when bound to SLAMF6 [90,91]. Essential for optimal GC responses by SLAMF5 binding [88].
CD40L	*CD40LG*	CD40	Essential for formation and maintenance of the GC, promotes antibody class switching and provides crucial survival signals to GC B cells [76,77,78,79].
EBI2	*GPR183*	7α,25-dihydroxycholesterol (7α,25-OHC)	Involved in temporospatial guidance of B cells and Tfh cells through the LN follicle mantle and GC [95].
S1PR1	*S1PR1*	S1P	Binds to S1P secreted by lymphatic vessels, facilitating T cell migration into the LN [33,34,35]. High concentrations of S1P in efferent lymph promotes Tfh cell egress from the LN via S1PR1 binding [50].
S1PR2	*S1PR2*	S1P	Involved in guidance and retention of Tfh cells within the GC via repelling them from S1P-rich lymph [95].
CD62L	*SELL*	GlyCAM-1	Facilitates adhesion and rolling of T cells along high endothelial venules as they migrate into the LN [36].
